# UAV Geo-Localization Dataset and Method Based on Cross-View Matching

**DOI:** 10.3390/s24216905

**Published:** 2024-10-28

**Authors:** Yuwen Yao, Cheng Sun, Tao Wang, Jianxing Yang, Enhui Zheng

**Affiliations:** School of Mechanical and Electrical Engineering, China Jiliang University, Hangzhou 310018, China; p22010854149@cjlu.edu.cn (Y.Y.); p22010854105@cjlu.edu.cn (C.S.); p23010854113@cjlu.edu.cn (T.W.); s22010811041@cjlu.edu.cn (J.Y.)

**Keywords:** UAV localization, geo-location datasets, satellite, deep learning

## Abstract

The stable flight of drones relies on Global Navigation Satellite Systems (GNSS). However, in complex environments, GNSS signals are prone to interference, leading to flight instability. Inspired by cross-view machine learning, this paper introduces the VDUAV dataset and designs the VRLM network architecture, opening new avenues for cross-view geolocation. First, to address the limitations of traditional datasets with limited scenarios, we propose the VDUAV dataset. By leveraging the virtual–real mapping of latitude and longitude coordinates, we establish a digital twin platform that incorporates 3D models of real-world environments. This platform facilitates the creation of the VDUAV dataset for cross-view drone localization, significantly reducing the cost of dataset production. Second, we introduce a new baseline model for cross-view matching, the Virtual Reality Localization Method (VRLM). The model uses FocalNet as its backbone and extracts multi-scale features from both drone and satellite images through two separate branches. These features are then fused using a Similarity Computation and Feature Fusion (SCFF) module. By applying a weighted fusion of multi-scale features, the model preserves critical distinguishing features in the images, leading to substantial improvements in both processing speed and localization accuracy. Experimental results demonstrate that the VRLM model outperforms FPI on the VDUAV dataset, achieving an accuracy increase to 83.35% on the MA@20 metric and a precision of 74.13% on the RDS metric.

## 1. Introduction

In recent years, the application of UAVs has expanded significantly in everyday life, including logistics delivery, bridge inspections, and security patrols [1,2]. UAV flights primarily rely on the Global Navigation Satellite System (GNSS) [3,4], but satellite signals are easily disrupted in battlefield environments or obstructed areas, causing instability in UAV flight. To address this issue, various auxiliary positioning methods have emerged, including SLAM technology [5], Inertial Navigation Systems (INSs) [6], and vision-based localization techniques [7,8,9]. While INS can provide continuous position information, it suffers from accumulated errors over prolonged operations. SLAM, although efficient, is limited by its need for real-time mapping and prior knowledge of the environment. Therefore, this study focuses on using vision-based methods to solve the localization challenge for UAVs operating in GNSS-denied environments [10]. The rapid advancements in deep learning for image processing have propelled significant progress in fields such as image retrieval [11], object tracking [12,13], and object detection [14,15], which, in turn, have driven the development and application of vision-based UAV localization techniques. These approaches overcome the limitations of traditional positioning technologies by leveraging rich image features and a sophisticated understanding of complex scenes, enhancing both accuracy and robustness in localization tasks.

Among the methods that use cross-view image matching to address UAV navigation in GNSS-denied environments, there are two primary approaches: retrieval-based methods [16] and direct image point-matching methods [17]. Retrieval-based approaches rely on building a massive image database, where UAV images are matched against satellite images in the database to estimate location. During training, metric learning is used to reduce the distance between UAV images and similar satellite images to improve location estimation. However, these methods require pre-collecting and constructing extensive image datasets, and mismatches can degrade localization accuracy, posing challenges for computational storage and processing. In contrast, Feature Point-based Inference (FPI) adapts concepts from object tracking, directly identifying corresponding locations on satellite images using UAV images, thereby reducing dependency on a satellite image database for cross-view matching. FPI employs a Siamese network with separate branches that extract features from UAV and satellite images without shared weights. By calculating the similarity between UAV and satellite feature maps, a heatmap is generated to map the UAV’s position onto the satellite image. However, this approach compresses feature information through multiple layers, leading to a significant loss of spatial details. Moreover, using only the final feature layer for similarity calculations can hinder localization accuracy. The datasets used in FPI are also limited, containing images from just 14 universities, which may limit the model’s generalization across diverse real-world scenarios, thus affecting practical localization accuracy.

Past datasets for cross-view matching research [18,19,20,21,22,23,24] have mostly focused on matching between street-view and aerial images or between street-view and satellite images. The introduction of the University-1652 dataset [25] provided a new perspective for cross-view geographic localization by incorporating UAV images, covering buildings and campus scenes. Subsequently, new datasets have been developed for UAV-based cross-view localization. For example, the DenseUAV dataset [26] sampled densely across 14 universities to avoid biases toward salient features, and the SUES-200 dataset [27] extended dense sampling to different altitudes, providing a novel approach for high-altitude UAV localization. Clearly, dataset construction is critical for UAV cross-view geographic localization. Traditionally, before testing a UAV localization algorithm, UAVs need to capture a large number of raw images that meet specific requirements to create corresponding datasets. This process consumes significant human resources and time, posing limitations on the broader adoption of UAV cross-view geographic localization methods in GNSS-denied environments.

Aiming at the above problems, this paper proposes a VDUAV dataset and designs a VRLM network, aiming to break the problem of a single dataset scene that is not easy to obtain, and, at the same time, optimize the network architecture to improve the positioning accuracy. At the dataset level, considering the advantages of digital twin technology, this paper imports the real scene model built by UAV using tilt photography technology in Unity3d, corrects the embedded model coordinates, performs coordinate mapping between the virtual platform and the real world through UTM projection, and finally builds a virtual platform that can simulate the image captured by the downward-looking camera of UAV in the real scene. The platform improves the production efficiency of the dataset and overcomes the defects of the previous dataset, which only focuses on mining salient features and high costs through the automated procedure of intensive sampling according to specific routes. The platform also expands the richness of the sampling scene, which can be switched to the 3D real-world model of different latitude and longitude areas, expands the richness and breadth of the dataset in the denial of the environment when UAVs carry out the cross-view geolocation algorithm, and finally obtains a dataset that includes cities. In the end, the VDUAV dataset containing multiple scenes, such as cities, plains, hills, factories, schools, rural areas, etc., is obtained, which ensures the diversity and comprehensiveness of the dataset and thus improves the generalization ability of the geolocation algorithm.

At the model level, a new cross-view matching network VRLM is proposed, which uses the first three stages of FocalNet as the feature extraction module of the model and extracts the multi-scale features of different stages of UAV images and satellite images through two branches. The shallow network can obtain more spatial information, while the deeper network learns more abstract semantic information and extracts the multi-scale features instead of outputting the final layer of feature information. The deeper network can synthesize the information contained in the image more comprehensively, which brings new ideas to the improvement of model performance. The obtained features are fed into the Similarity Computing Feature Fusion (SCFF) module, which uses an adaptive approach to the weighted fusion of multi-scale features, aiming at retaining the key distinguishing features of UAV images and satellite images in a more complete way.

The main contributions of this paper are as follows:

1. Utilizing the advantages of digital twin technology, the construction of the VDUAV dataset solves the problems of the traditional cross-view matching dataset with a single scene and high production cost.
2. A new UAV cross-view geo-localization model, VRLM, is proposed, which performs multi-scale feature extraction on UAV images and satellite images through the first three stages of the FocalNet backbone network. This effectively mitigates the serious loss of image position information due to multiple compression and designs an adaptive multi-scale feature weighted fusion module (SCFF) to maximize the retention of image information; this improves the running speed and positioning accuracy of the model.
3. Based on the VDUAV dataset and model proposed in this paper, using RDS as an evaluation metric, the model accuracy is improved from 67.07% to 74.13% compared with FPI, and the model achieves 45.13%, 64.71%, and 83.35% localization accuracies at the 5 m, 10 m, and 20 m levels, respectively, when evaluated with the meter-level accuracy rubric.

The remainder of this paper is organized as follows: Section 2 reviews related work. Section 3 describes the construction of the digital twin platform. Section 4 introduces the VDUAV dataset. Section 5 details the proposed VRLM baseline model. Section 6 presents comparative experiments with the FPI geographic localization method. Section 7 provides the results of ablation studies, and Section 8 concludes the paper.

## 2. Related Work

### 2.1. Geo-Location Datasets

In the context of drone self-localization in denied environments, the construction of the datasets plays a crucial role in determining the accuracy of localization. Early studies primarily relied on using publicly available maps and aerial images to create image pairs [28]. Subsequently, the CVUSA dataset [29] introduced a method for matching ground-level panoramic images from different regions in the United States with satellite images. The VIGOR dataset [24] further improved retrieval accuracy by adjusting the search area of the query images, while the University-1652 dataset [25] advanced drone geolocation to a new stage by matching drone views with satellite images, significantly enhancing the accuracy of drone self-localization.

Recent years have seen continued progress in research on matching drone views with satellite images for geolocation. The DenseUAV dataset [26] introduced dense sampling for drones, where adjacent images have overlapping regions, while also considering the characteristic information and spatial layout of buildings. The SUES-200 dataset [27] provided images from multiple different altitudes in the drone’s perspective, optimizing the model’s compatibility across different flight heights. The GLVL method [30] improved the accuracy of drone localization by matching downward-facing drone images with remote sensing images, considering the influence of feature vector size and performing orthogonal correction. The UL14 dataset [17], built on DenseUAV, transforms the task from image retrieval to image matching, further enhancing the drone’s self-localization capabilities. The UAV-VisLoc dataset [31] introduced a method for data collection across multiple angles and scenes, increasing the diversity of the dataset and improving the robustness and generalization of localization algorithms. Table 1 summarizes the existing geolocation datasets, detailing aspects such as sampling environments, dataset sizes, and evaluation metrics.

### 2.2. Deep Learning-Based Geo-Localization Method for UAV

In this paper, we focus on matching and localizing between drone images and satellite images. With the advancement of deep learning technologies and the increasing application of drones, many methods have emerged based on drone vision for geolocation.

Ding et al. [32] proposed a cross-view matching method based on location classification, which simplifies the retrieval problem into a classification problem. This approach considers the impact of feature vector size on matching accuracy, enabling bidirectional matching between drone images and satellite images. Subsequently, Tian et al. [33] utilized Perspective Projection Transformation (PPT) and Conditional Generative Adversarial Networks (CGANs) to synthesize drone images that closely resemble real satellite images. They proposed an end-to-end UAV–satellite cross-view geolocation method. Mughal et al. [34] introduced a complete end-to-end trainable architecture that applies probabilistic constraints to dense correlation feature maps across different dimensions. This method simultaneously performs feature learning and template localization, enhancing the matching accuracy of drone images. Cui et al. [35] proposed a single-stage image retrieval method, designing a segmented soft-margin triplet loss function. This function effectively avoids the issue of the model parameters being trapped in a suboptimal set due to the lack of constraints on positive and negative samples, thereby improving the accuracy of image retrieval and achieving better convergence. Dai et al. [26] suggested comparing the similarity between drone images and satellite images in vertical views by calculating the cosine distance between them. The method then determines the most similar image by calculating the cosine distance between the drone image and the satellite image database, thus achieving drone localization and navigation.

These methods collectively contribute to advancing the field of cross-view geolocation by improving the accuracy and efficiency of matching drone images with satellite images, thereby enabling more robust and reliable drone self-localization in denied environments.

### 2.3. Transformer

The Transformer model, initially introduced for natural language processing [36,37], has achieved remarkable success in tasks such as machine translation. Subsequent research revealed that the attention mechanism of Transformers [38] also performs well in image data processing. Traditional Convolutional Neural Networks (CNNs) [39] have been fundamental in image processing tasks, but they have limitations in handling global context information and long-term dependencies. The introduction of Vision Transformer (ViT) [40] addressed these limitations by segmenting images into sequences and embedding positional information. This approach leverages the self-attention mechanism in Transformers to capture global context and better handle dependencies. Touvron et al. proposed the Deit model [41], which achieved efficient classification on small-scale datasets through knowledge distillation. Later, Wang et al. introduced the PVT model [42,43], incorporating a multi-scale design with parallel pyramid structures to capture features at different scales.

Recent advancements in Transformer models for visual target tracking have been significant. Transtrack [44] introduced a multi-channel self-attention mechanism to model video sequences, improving target tracking accuracy by establishing global target–background relationships. TransT [45] utilized multi-layer Transformer encoders to extract feature representations from video sequences, effectively modeling spatial and temporal relationships of targets for continuous tracking. Wang [46] applied this structure to the domain of drone cross-view geolocation, using a dual-stream network with shared weights to extract features from drone and satellite images. This approach predicts the relative positions and optimizes the backbone network, enhancing the accuracy of drone geolocation in denied environments.

## 3. Digital Twin Platform Building

The digital twin platform was constructed through two main processes: 3D modeling and coordinate mapping. For this setup, we selected a specific area in Zhejiang Province, China, using a 1:500 scale topographic map for the survey. We captured the terrain of the experimental area from multiple angles using oblique photogrammetry techniques. These images were then processed using ContextCapture software 10.20 to create a 3D realistic model in OSGB format. The OSGB model was imported into Unity3D, where it was integrated into a virtual environment. We used the transverse Mercator projection method to map the model’s coordinates, aligning the Cartesian coordinates in Unity3D with the WGS84 latitude and longitude coordinates of the physical space.

Additionally, by accessing a wide range of pre-established 3D digital twin models through public channels, we were able to enhance the dataset’s diversity and accuracy, ensuring that it includes various regions and scenes for future applications.

### 3.1. UAV Tilt-Photography Modeling

**Figure 1:**
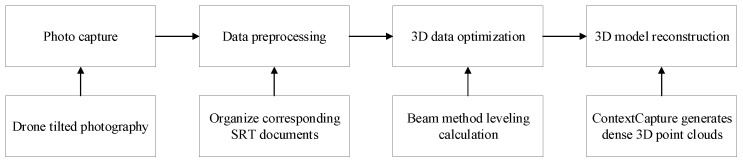
Flowchart of UAV tilt-photography modeling.

Oblique photography technology, a shining star in the field of surveying, has seen rapid development in recent years. To efficiently and accurately recreate the 3D scene of the flight area, this paper uses advanced UAV oblique photography technology for comprehensive real-world modeling. The technology involves capturing surface images from five directions—vertical and four oblique angles (front, back, left, and right)—using a multi-angle camera system mounted on the UAV. By setting up ground control points within the survey area, the aerial images are oriented and transformed into coordinates. The measurement results are unified and computed within the WGS84 coordinate system, enabling rapid 3D reconstruction of large areas, as illustrated in Figure 1, which shows the UAV oblique photography modeling process.

1. **Pre-Flight Setup:** Data collection was conducted using a DJI M210 drone (Shenzhen DJI Innovation Technology Co., LTD., Shenzhen, China). Prior to takeoff, the reconstruction area was defined, and the drone’s flight path was automatically planned based on the required overlap rate to ensure comprehensive coverage. During the flight, an SRT file containing time-synchronized data was recorded. Exposure compensation and other parameters were adjusted to ensure image clarity.
2. **Data Preprocessing:** The raw video and corresponding SRT files were organized. The video was exported frame by frame into images, and waypoint data from the SRT files were embedded into the corresponding image metadata. This step included the calibration of the drone camera’s internal parameters.
3. **The 3D Data Optimization:** The processed camera calibration data and image data were imported into Metashape software 2.0.4 for feature point extraction from images taken from five different viewpoints. Bundle adjustment was employed to perform overall adjustment calculations, removing gross error points. Through multiple iterations of bundle adjustment and point position adjustments, the optimized camera poses for each image were obtained.
4. **The 3D Model Construction:** The collected images and processed data were imported into ContextCapture software [47]. The optical properties, including sensor dimensions and focal length information, are also imported. After verifying that the information is correct, image matching is performed. Once matching is completed, aerial triangulation is conducted, and ground control points are imported. A manual association of ground control points with images is carried out, and the ground control points are manually adjusted. Finally, aerial triangulation measurement is performed, and once verified, a 3D model and orthophoto are generated.

### 3.2. Model Import

Integrating the OSGB 3D reality model constructed through UAV oblique photogrammetry into the digital twin platform not only enhances the realism of virtual space visualization but also facilitates the subsequent capture and creation of UAV localization datasets in denied environments.

**Figure 2:**
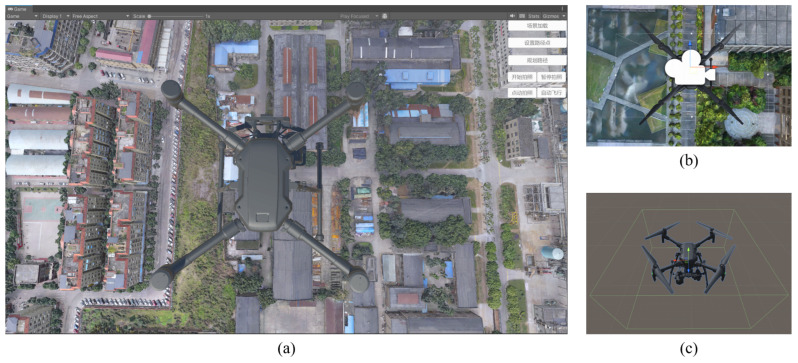
(**a**) The drone geolocation system built by importing the processed real-world 3D model into the Unity3D engine using Digital Twin technology. (**b**) The virtual camera model. (**c**) The virtual drone collision test model.

First, the constructed OSGB 3D reality model is integrated into Unity3D, as shown in Figure 2a, along with the embedded UAV model. The virtual UAV simulation model includes basic geometric shapes, physical properties, and sensor models. The 3D model of the UAV is created using SolidWorks and imported into Unity. Next, a virtual camera and physical collision model are added to the UAV within Unity. The virtual camera of the UAV, illustrated in Figure 2b, uses Unity’s camera component to simulate the onboard camera. This component allows for the configuration of the virtual camera’s mounting angle and field of view to accurately replicate the actual UAV camera setup. Specifically, by adjusting the virtual camera’s mounting angle and field of view, various visual effects under different flight conditions can be simulated, providing realistic sampling data for subsequent UAV geographic localization algorithms.

The UAV’s collision model, shown in Figure 2c, is simplified to a rectangular bounding box that encompasses the actual dimensions of the UAV. This simplification enhances the efficiency of simulation computations while maintaining accuracy in collision detection.

### 3.3. Accurate Mapping of Real and Virtual Space

Drones obtain absolute position information based on latitude and longitude coordinates in physical space, while virtual space uses a Cartesian coordinate system. To achieve accurate mapping between these two systems, this paper adopts the Universal Transverse Mercator (UTM) projection method for coordinate transformation between physical and virtual spaces.

The UTM projection divides the Earth into 60 zones, each covering 6° of longitude and 8° of latitude, and is a conformal transverse cylindrical projection. This paper performs UTM projection transformations based on the WGS84 ellipsoid, converting geographic length units to meters and geographic coordinate units to radians. This method effectively maps the drone’s latitude and longitude coordinates from physical space to the Cartesian coordinate system in virtual space, enhancing the accuracy and reliability of the virtual–physical integration.

To convert latitude and longitude coordinates to planar Cartesian coordinates, the forward UTM projection formulas are used:

(1) $$\left\{\begin{array}{c} x=FE+k_{0}\left(Ncl+\frac{N}{6}c^{3}l_{3}l^{3}+\frac{N}{120}c^{5}l_{5}l^{5}+\frac{N}{5040}c^{7}l_{7}l^{7}\right)\\ y=FN+k_{0}\left(M+\frac{Nt}{2}c^{2}l^{2}+\frac{Nt}{24}c^{4}l_{4}l^{4}+\frac{Nt}{720}c^{6}l_{6}l^{6}+\frac{Nt}{40320}c^{8}l_{8}l^{8}\right) \end{array}\right.$$

where FN and FE represent coordinate offsets, with FE being 0 in the Northern Hemisphere and FN being 10,000,000 in the Southern Hemisphere. The parameter $k_{0}$ is the scale factor, while *N* denotes the radius of curvature in the prime vertical. The variable ϕ represents latitude, and *c* is the cosine of the latitude. The term *M* signifies the meridian arc length from the equator to the given latitude ϕ. Additionally, $l_{3}$, $l_{4}$, $l_{5}$, $l_{6}$, $l_{7}$, and $l_{8}$ are coefficients corresponding to their respective terms in the equations.

To convert Cartesian coordinates back to latitude and longitude, the inverse UTM projection formulas are used:

(2) $$\left\{\begin{array}{c} \phi =\phi_{f}+\frac{t_{f}}{2N_{f}^{2}}x_{2}\Delta x^{2}+\frac{t_{f}}{24N_{f}^{4}}x_{4}\Delta x^{4}+\frac{t_{f}}{720N_{f}^{6}}x_{6}\Delta x^{6}+\frac{t_{f}}{40320N_{f}^{8}}x_{8}\Delta x^{8}\\ \lambda =\lambda_{0}+\frac{1}{N_{f}c_{f}}\Delta x+\frac{1}{6N_{f}^{3}c_{f}}x_{3}\Delta x^{3}+\frac{1}{120N_{f}^{5}c_{f}}x_{5}\Delta x^{5}+\frac{1}{5040N_{f}^{7}c_{f}}x_{7}\Delta x^{7} \end{array}\right.$$

where parameters with the subscript *f* need to be calculated based on the latitude of the bottom point $\phi_{f}$, and Δx, $x_{2}$, $x_{3}$, $x_{4}$, $x_{5}$, $x_{6}$, $x_{7}$, $x_{8}$, and $\phi_{f}$ are computed as follows:

(3) $$\left\{\begin{array}{c} \Delta x=\frac{x-FE}{k_{0}}\\ x_{2}=-1-\nu_{f}^{2}\\ x_{3}=-1-2t_{f}^{2}-\nu_{f}^{2}\\ x_{4}=5+3t_{f}^{2}+6\nu_{f}^{2}-6t_{f}^{2}\nu_{f}^{2}-3\nu_{f}^{4}-9t_{f}^{2}\nu_{f}^{4}\\ x_{5}=5+28t_{f}^{2}+24t_{f}^{4}+6\nu_{f}^{2}+8t_{f}^{2}\nu_{f}^{2}\\ x_{6}=-61-90t_{f}^{2}-45t_{f}^{4}-107\nu_{f}^{2}+162t_{f}^{2}\nu_{f}^{2}+45t_{f}^{4}\nu_{f}^{2}\\ x_{7}=-61-662t_{f}^{2}-1320t_{f}^{4}-720t_{f}^{6}\\ x_{8}=1385+3633t_{f}^{2}+4095t_{f}^{4}+1575t_{f}^{6}\\ \phi_{f}=\bar{y}+\bar{\beta }\sin\left(2\bar{y}\right)+\bar{\gamma }\sin\left(4\bar{y}\right)+\bar{\delta }\sin\left(6\bar{y}\right)+\bar{\varepsilon }\sin\left(8\bar{y}\right) \end{array}\right.$$

This study not only integrates autonomously collected drone oblique photography models but also includes publicly available real-scene oblique photography models to enrich the dataset’s content and coverage. Various regions with a wide range of latitudes and multiple terrain features were selected to enhance the dataset’s diversity and comprehensiveness, ensuring a thorough assessment and improvement of the system’s positioning accuracy.

To verify the consistency of the mapping between virtual and real-world location points, we randomly selected nine reference points within the virtual environment. Subsequently, the latitude and longitude coordinates of these reference points were measured in the field using high-precision Real-Time Kinematic (RTK) technology. These coordinates were then converted to 3D coordinates using the UTM projection transformation formulas and compared with the 3D coordinates of the selected positions in the virtual space to evaluate the mapping accuracy.

**Figure 3:**
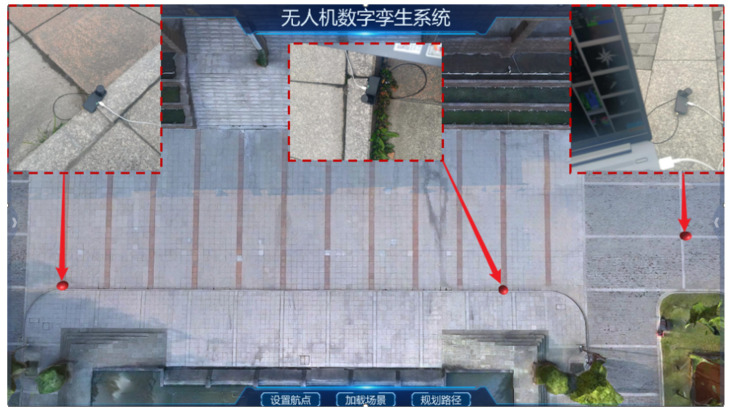
RTK field measurements.

By quantifying the discrepancies between the two sets of coordinates, we assessed the accuracy of the coordinate mapping. The red dots in Figure 3 represent the locations in the virtual space corresponding to the latitude and longitude coordinates of the physical reference points. The photos within the dashed boxes show the positions measured by the RTK device in the physical space.

**Figure 4:**
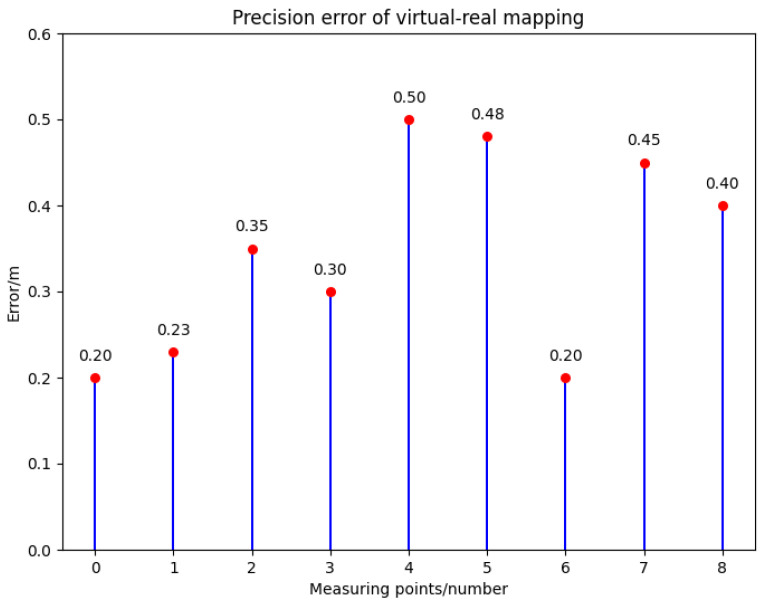
Virtual reality mapping error analysis of nine arbitrarily selected measurement points on a solid model of Hangzhou, China, measured by RTK.

The error distribution of the nine selected reference points is shown in Figure 4. The maximum error was 0.51 m, the minimum error was 0.19 m, and the average mapping error across the test samples was 0.38 m. This error could affect the creation of the drone dataset in the virtual environment. Therefore, a self-checking mechanism was added to the digital twin platform, excluding areas with errors greater than 0.2 m from the drone dataset collection scenes. By implementing this self-checking mechanism, the reliability and accuracy of the virtual dataset creation were improved, reducing the deviations between the virtual and real environments.

## 4. VDUAV Dataset

### 4.1. Dataset Creation

In Section 3.1, the established digital twin platform innovatively employs the UAV downward-facing camera for image acquisition within the virtual space. This approach differs from the methods used for data collection in previous datasets such as DenseUAV and SUES-200. The dataset proposed in this study represents a pioneering integration of digital twin technology with UAV geographic localization. It not only retains the advantages of dense and multi-altitude sampling datasets but also incorporates a wide range of 3D models from various geographic locations through publicly available sources. Compared with traditional dataset creation methods, these publicly sourced models are simpler and faster to obtain, enhancing the dataset’s diversity and geographic coverage. This approach aims to ensure the richness and accuracy of the dataset while minimizing the consumption of time and resources.

**Figure 5:**
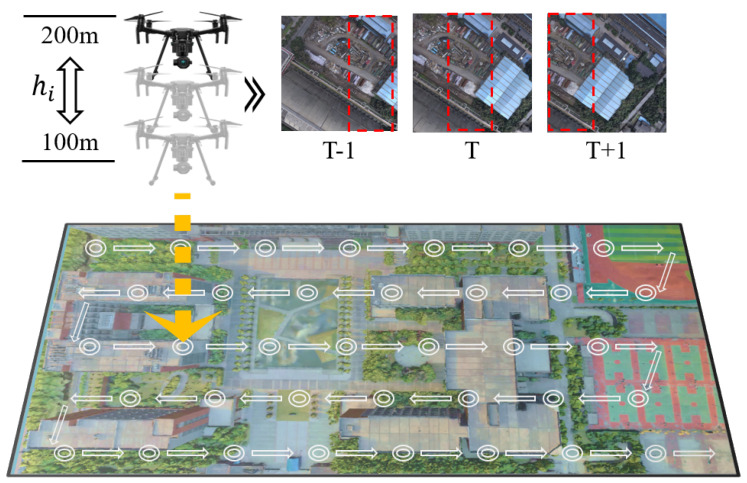
The construction process of the VDUAV dataset, which involves the UAV capturing images every 20–30 m at each point; images are taken at three different heights ranging between 100 and 200 m.

During the construction of the training set, the downward-facing camera of the drone in the digital twin platform was used to photograph the ground vertically, employing a dense sampling method for data collection. Unlike previous dataset creation methods, the flexibility in controlling drone flight within the digital twin platform allowed us to introduce an interval adjustment parameter δ, enabling the data collection interval to randomly vary between 20 m and 30 m. For the vertical dimension, we introduced a height adjustment parameter η for each selected shooting position, allowing random selection of three different altitudes between 100 m and 200 m above sea level. The specific construction process of the VDUAV dataset is shown in Figure 5, and Table 2 details the multi-scene data collection in different regions. The multi-level dense sampling requires the model to capture fine-grained features during training and understand spatial information, enhancing the model’s ability to capture and understand complex three-dimensional spatial information.

(4) $$\left\{\begin{array}{c} d=\delta \times 20,\;\forall \delta \in (1,1.5)\\ h_{i}=\eta_{i}\times 100,\;i=1,2,3\;\forall \eta_{i}\in \left(1,2\right) \end{array}\right.$$

where *d* is the interval distance for data acquisition in the virtual environment, δ is the interval adjustment parameter, $h_{i}$ represents the three flight altitudes selected at a waypoint, and $\eta_{i}$ is the height adjustment parameter used to adjust the drone’s flight altitude.

**Figure 6:**
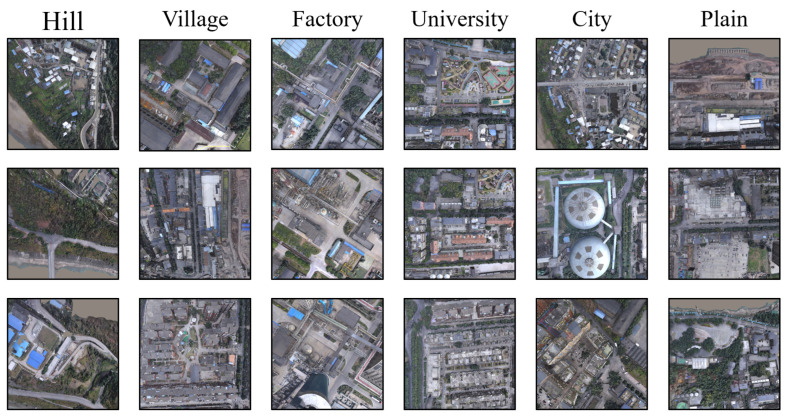
Drone images collected from various scenarios, including urban areas, village regions, hilly terrains, plains, university campuses, and industrial sites.

Most notably, all processes for completing drone image acquisition were carried out within the digital twin platform, significantly saving the time and resources required for real-world data collection. Drone images collected in multiple scenarios are shown in Figure 6.

To ensure the accuracy of the satellite images, we collected them using level 20 tiles from Google Maps. This approach provides high-resolution and clear satellite images, allowing for a detailed display of geographic features and terrain characteristics.

### 4.2. Drone–Satellite Map Data Pairs

**Training Set Creation:** Images from various scenes in the digital twin platform were captured at different ranges and heights. GPS latitude and longitude information, calculated from the virtual–real mapping, was embedded in each image’s EXIF file. Using this GPS information, the corresponding drone capture points were located on pre-cut satellite maps to create drone–satellite image pairs. Drone images in the training set were center-cropped to a resolution of 256 × 256 × 3. Using the latitude and longitude information stored in the EXIF files, the corresponding satellite images were quickly identified, and their resolution was standardized to 1280 × 1280 × 3.

**Test Set Creation:** For the test dataset, we selected drone images from urban areas, plains, hills, factories, and schools, ensuring no overlap with the training set. Corresponding satellite images were identified based on the GPS coordinates of the drone images. The satellite images for the test set were standardized to a resolution of 384 × 384 × 3. Unlike the training set, we applied data augmentation to the satellite images in the test set. Specifically, we selected satellite image ranges at 100-pixel intervals, from 700 pixels to 1800 pixels, creating 12 different scales of satellite images. The 700-pixel scale corresponds to a physical space dimension of approximately 180 m × 180 m, while the 1800-pixel scale corresponds to approximately 463 m × 463 m.

**Figure 7:**
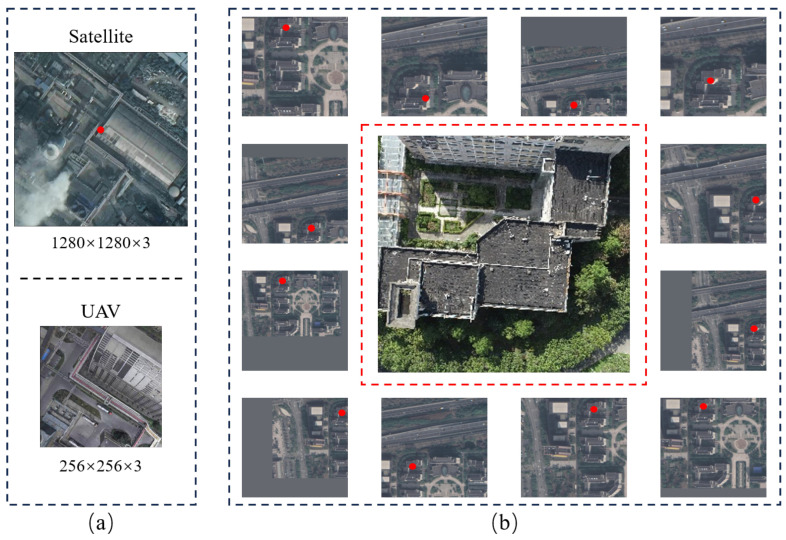
(**a**) The training set, where the ratio of drone images to satellite images is 1:1. (**b**) The test set, where drone images are enclosed in red dashed boxes, and satellite images are enclosed in black dashed boxes, with a ratio of 1:12. The solid red dots in the images indicate the actual positions of the drones in the satellite images.

Figure 7 illustrates the dataset configuration. Figure 7a shows the training set with a 1:1 ratio of drone images to satellite images. Figure 7b depicts the test set with a 1:12 ratio, where drone images are enclosed in red dashed boxes, and satellite imagery is included between the red dotted line and the black dotted line. The red solid dots indicate the true locations of the drones in the satellite images. Table 3 shows the distribution of drone and satellite images in the training and test sets.

To ensure precise alignment between drone and satellite images, the drone images were cropped to match the geometric centers of the corresponding satellite images. To better train the model and address image discrepancies caused by temporal shifts, data augmentation was applied. Satellite images were dynamically cropped to various scales, with excess areas filled with average pixel values, as shown by the gray-filled portions in Figure 7b. This approach aims to improve the model’s generalization capabilities.

## 5. Methods

In this chapter, we will provide a detailed introduction to the geographic localization algorithm framework and the specifics of each module. Section 5.1 presents the overall framework of the VRLM localization algorithm, which achieves drone localization through feature extraction, feature fusion, and heatmap generation. Section 5.2 explains the advantages of the FocalNet backbone network. Inspired by Transformer networks, FocalNet incorporates focus modulation and context aggregation modules for more refined image feature extraction. Section 5.3 details the Similarity Calculation Feature Fusion module (SCFF), which computes and merges features extracted by the dual-stream backbone network. This fusion of shallow spatial information and deep semantic information significantly enhances the algorithm’s localization accuracy.

### 5.1. Deep Learning Modeling Framework

**Figure 8:**
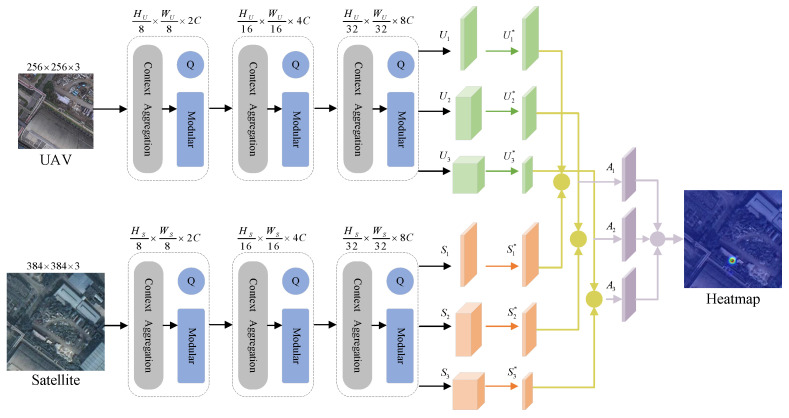
The VRLM fusion localization network framework first performs feature extraction to obtain two sets of features, $U_{1}$, $U_{2}$, $U_{3}$ and $S_{1}$, $S_{2}$, $S_{3}$. Subsequently, the similarity between the two sets of features is calculated, represented by yellow spheres. The purple spheres represent weighted fusion, ultimately generating a heatmap.

In this section, we introduce the framework of the VRLM-fused geographic localization model, as illustrated in Figure 8. This network adopts a structure similar to the Siamese dual-stream network for feature extraction between drone and satellite images. However, unlike traditional methods, using a weight-sharing network might not be beneficial due to the significant differences between drone and satellite images.

We chose to input 256 × 256 × 3 drone images and 384 × 384 × 3 satellite images into two networks without shared weights. FocalNet is used as the backbone network for feature extraction from both sets of images. Inspired by the attention mechanism of Transformer networks, FocalNet incorporates the Context Aggregation Module (CAM) and Focal Modulation Module (FMM), enabling the network to capture both local and global feature information, thereby improving the model’s performance.

The extracted features from the three stages are fed into the Similarity Calculation and Feature Fusion (SCFF) module. This module computes the similarity between the downscaled drone and satellite images to the same dimension and derives their adaptive weights. Finally, the features are weighted and fused to generate a heatmap with precise location information, which is then mapped back onto the satellite image.

### 5.2. Backbone Network

The drone images (denoted as *U*, with dimensions $W_{U}\times H_{U}\times 3$) and satellite images (denoted as *S*, with dimensions $W_{S}\times H_{S}\times 3$) are input into the FocalNet network. We choose the first three stages of this network for feature extraction, with each stage consisting of 2, 2, and 6 layers, respectively. After processing through each stage, the output image resolution is halved in both height *H* and width *W*, and the number of channels is adjusted to 192, 384, and 768. Specifically, the outputs are $\frac{H}{8}\times \frac{W}{8}\times 2C$, $\frac{H}{16}\times \frac{W}{16}\times 4C$, and $\frac{H}{32}\times \frac{W}{32}\times 8C$. Each stage repeats a similar structure: first, a convolutional layer extracts the initial feature map, which then enters the Focus Modulation Module (FMM). FMM selectively enhances features in regions of interest using a local attention mechanism, calculates the focus modulation weights, and applies them to the feature map, allowing the network to concentrate more on significant areas. Next, the Context Aggregation Module (CAM) enhances features from a global perspective, supplementing the fine-grained information extracted by FMM. CAM aggregates contextual information from the entire feature map through a global attention mechanism, computes weights, and fuses the global context information with the fine-grained features. After a series of processes, the final feature maps are obtained as 12 × 12 × 768 and 8 × 8 × 768.

Inspired by the self-attention mechanism in Transformer networks, FocalNet introduces the Focus Modulation Module and Context Aggregation Module. These modules not only enable the dynamic weighting of features through the “focus” mechanism but also capture long-range dependencies, retaining critical information while reducing computational complexity. This helps the network better understand the semantic information of input images and enhances its ability to comprehend complex visual scenes.

### 5.3. Similarity Computation Multi-Feature Fusion Module

FocalNet extracts features at different scales across three stages. We observed that the shallow layers of the network contain more spatial feature information, while the deeper layers contain more semantic information. By utilizing the Similarity Calculation Feature Fusion (SCFF) module, we dynamically select and fuse multi-level features to better capture multi-scale target information. The extracted feature maps are first dimensionally reduced to unify their dimensions. Then, we calculate the similarity between the drone and satellite feature maps, pair by pair. An adaptive weighting mechanism fuses the information from these similarity-calculated feature maps. This fusion preserves the advantages of multi-scale features and reduces feature redundancy through spatial weighting, thus enhancing the accuracy of the algorithm.

Taking the three sets of features $\left\{U1,\;U2,\;U3\right\}$ extracted from the drone branch as an example, we first use a 1 × 1 convolution kernel to adjust the channel numbers and unify the dimensions of the feature maps, resulting in $\left\{{U1}^{*},{U2}^{*},{U3}^{*}\right\}$. The processed drone and satellite feature maps are then calculated using cosine similarity to obtain A1, A2, A3. Next, we employ a 1 × 1 convolution kernel and a softmax function for adaptive weight learning, yielding the spatial weight parameters $\alpha_{ij},\beta_{ij},\gamma_{ij}$, which are normalized. Finally, features are weighted and fused according to these weights, preserving the strengths of multi-scale features while effectively integrating them through the adaptive weighting mechanism.

(5) $$x_{ij}^{l}=\alpha_{ij}^{l}\cdot A_{ij}^{1\to l}+\beta_{ij}^{l}\cdot A_{ij}^{2\to l}+\gamma_{ij}^{l}\cdot A_{ij}^{3\to l}$$

(6) $$\left\{\begin{array}{c} \alpha_{ij}^{l}=\frac{e^{\lambda \alpha_{ij}}}{e^{\lambda \alpha_{ij}}+e^{\lambda \beta_{ij}}+e^{\lambda \gamma_{ij}}}\\ \beta_{ij}^{l}=\frac{e^{\lambda \beta_{ij}}}{e^{\lambda \alpha_{ij}}+e^{\lambda \beta_{ij}}+e^{\lambda \gamma_{ij}}}\\ \gamma_{ij}^{l}=\frac{e^{\lambda \gamma_{ij}}}{e^{\lambda \alpha_{ij}}+e^{\lambda \beta_{ij}}+e^{\lambda \gamma_{ij}}} \end{array}\right.$$

Here, $u_{ij}^{1\to l}$, $u_{ij}^{2\to l}$, and $u_{ij}^{3\to l}$ represent the values of feature maps A1, A2, and A3 after being upsampled and aligned to the same resolution. $\alpha_{ij}^{l}$, $\beta_{ij}^{l}$, and $\gamma_{ij}^{l}$ are the adaptive weight parameters, indicating the weights of the feature maps at position (i,j), with λ as a tuning parameter to control the smoothness of the weights.

## 6. Experiment

### 6.1. Experimental Details

The training process was conducted on an NVIDIA 1080TI GPU, using PyTorch 1.10.2 and Python 3.7 as the software environment. The training parameters included a batch size of 16, 32 epochs, and a learning rate set to 0.0001. FocalNet was selected as the backbone network for the VRLM model, utilizing publicly available pre-trained weights.

Drone images were standardized to a size of 256 × 256 × 3, while satellite images were adjusted to 384 × 384 × 3. The dual-stream branches of the network extracted features from the first three stages, with depths of 2 layers, 2 layers, and 6 layers, respectively. These extracted features were then input into the Similarity Computation Feature Fusion module for fusion.

### 6.2. Datasets and Evaluation Indicators

This study uses the VDUAV dataset, which combines the dense sampling characteristics of the DenseUAV dataset, where overlapping regions exist between adjacent frames to enhance the model’s ability to capture fine-grained features. It also incorporates the varied altitude sampling characteristics of the SUES-200 dataset, enhancing the model’s ability to recognize relevant spatial information. The dataset includes diverse scenarios with significant differences in building characteristics, such as rural areas, cities, campuses, hills, and factories, posing a challenge to the robustness and generalization capability of the localization model. The training set consists of 9265 drone images and an equal number of satellite images. The test set features data augmentation for the satellite images, with a ratio of 1:12 between drone and satellite images. This comprehensive dataset enables the model to better handle various complex real-world scenarios.

To ensure fairness, the same evaluation metrics as FPI are adopted: Meter-level Accuracy (MA@K) and Relative Distance Score (RDS). MA@K assesses the deviation from the actual geographic location, while RDS evaluates the pixel deviation in satellite images. The formula for MA@K is:

(7) $$\mathrm{MA}@K=\frac{1}{N}\sum_{i=1}^{N}I_{i}$$

where $e_{i}$ is the localization error in geographic space for the *i*-th sample. The accuracy $I_{i}$ for a single sample is determined based on whether its spatial localization error is within the threshold *K*.

Here, *K* is an adjustable parameter representing the real spatial distance in meters, and *N* is the number of test samples in the dataset. MA@K intuitively reflects the model’s performance by calculating the accuracy and latitude distance between the actual and predicted positions. The formula for RDS is:

(8) $$RDS=e^{-k\times \sqrt{\frac{{\left(\frac{dx}{w}\right)}^{2}+{\left(\frac{dy}{h}\right)}^{2}}{2}}}$$

where *w* represents the pixel width of the satellite image, *h* represents the pixel height, dx is the pixel distance between the predicted and actual horizontal coordinates, and dy is the pixel distance between the predicted and actual vertical coordinates. The scaling factor *k* is set to 10. RDS provides a more comprehensive assessment of model performance by evaluating the pixel-level accuracy of the predicted positions.

### 6.3. Main Results

#### 6.3.1. Comparative Analysis of Positioning Methods

**Figure 9:**
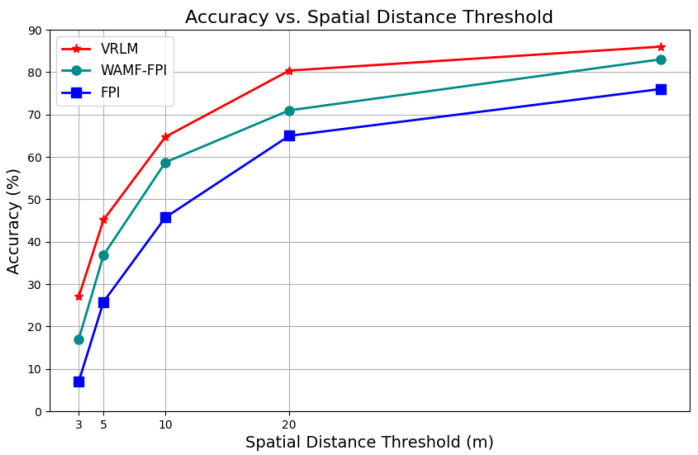
The localization accuracy of FPI, WAMF-FPI, and VRLM using the MA evaluation metric.

To ensure the rigor and comprehensiveness of the experimental results, we compared the proposed VRLM geographic localization method with mainstream image-matching localization methods (FPI and WAMF-FPI) on the same VDUAV dataset. Table 4 presents the RDS scores, parameter counts, computational operations, and meter-level precision across different thresholds for each model. It is evident that VRLM reduces both parameter complexity and computational load compared to FPI and WAMF-FPI. After feeding 256 × 256 × 3 UAV images and 384 × 384 × 3 satellite images into the network, the experimental results highlight the superior performance of VRLM. At lower GFLOPS, VRLM achieved a 7.06-point improvement in comprehensive RDS scores compared to FPI, and its localization performance also showed significant advantages over WAMF-FPI. Additionally, we evaluated the meter-level localization precision of VRLM, as illustrated in Figure 9. Compared to FPI, our model demonstrated consistent improvements across various distance thresholds (5 m, 10 m, and 20 m), with precision gains of 13.32%, 12%, and 11.38%, respectively.

This may be due to the loss of feature information due to multiple compression of the image feature output from the last layer of the backbone network in FPI, which is noted in WAMF-FPI, which modifies the model structure so that the model outputs feature information at each stage, followed by multi-feature fusion, which leads to an improvement in the model’s RDS metric scores and the meter-level accuracy localization results. However, its PCPVT network uses a fixed multi-scale pyramid feature extraction method, which lacks flexibility in capturing local and global information and consumes too many computational parameters and computational speed, greatly reducing the speed of model inference. At the same time, the UL14 dataset used by FPI and WAMF-FPI consists of only 14 colleges and universities in Zhejiang Province, and the model trained from this dataset lacks generalization ability, and its localization accuracy will be greatly reduced if the FPI and WAMF-FPI models are used to validate the areas with large differences in architectural structures, such as rural areas, urban areas, and plains. In this paper, the first three stages of the FocalNet network are used to replace the feature extraction module in WAMF-FPI for multi-feature extraction. The focal mechanism has a more efficient feature aggregation ability so that the network can focus on the local features while also integrating the global information appropriately. There is a more efficient ability to capture the details and connect the context in complex scenes, which improves the model’s sensitivity to the complex image and detail sensitivity of the model to complex images and changes in details. Moreover, the focal mechanism can extract key features when the dataset changes significantly (e.g., scene, lighting, viewpoint, etc.) through flexible feature selection, which enhances the generalization ability of the model. This is especially important when training datasets containing multiple geographic environments.

At the same time, a multi-feature weighted fusion structure, SCFF, is designed. Compared with FPI, which directly performs similarity computation on the last feature map, the low-resolution feature maps directly affect the final accuracy of the model. The SCFF module first uses weighted fusion to fuse the multi-scale feature maps generated in the first three phases of the FocalNet network so that the features of similarity computation not only include the high-resolution feature maps but also the spatial information of the horizontal feature maps and the spatial information of the horizontal feature maps. The features for similarity calculation not only contain the spatial information of the high-resolution feature maps but also fuse the deeper feature maps with rich semantic information through the horizontal connection structure, which speeds up the computation speed of the model and improves the accuracy of the localization algorithm at the same time.

**Figure 10:**
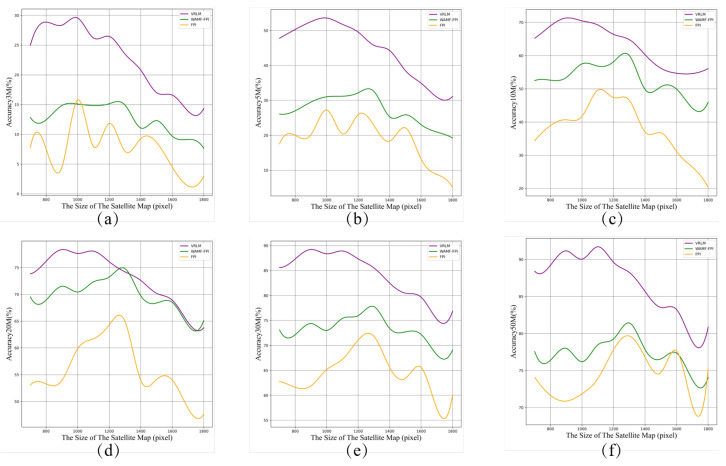
Comparing the performance of different models on varying satellite map sizes, with the *x*-axis representing the scale of the satellite images and the *y*-axis representing the localization accuracy of the models at the corresponding level. (**a**) Localization accuracy at 3 m. (**b**) Localization accuracy at 5 m. (**c**) Localization accuracy at 10 m. (**d**) Localization accuracy at 20 m. (**e**) Localization accuracy at 30 m. (**f**) Localization accuracy at 50 m.

We further evaluated the model’s performance on satellite maps of varying scales (ranging from 700 pixels to 1800 pixels). As illustrated in Figure 10, the performance of FPI, WAMF-FPI, and VRLM was analyzed across six images, each representing the model’s localization accuracy at different satellite image scales for 5 m, 10 m, 20 m, 30 m, 40 m, and 50 m accuracy levels. The *x*-axis indicates the scale of the satellite images, while the *y*-axis shows the corresponding localization accuracy at each level. The results demonstrate that the VRLM model consistently outperformed both FPI and WAMF-FPI across all scales of satellite maps, highlighting its robustness and generalization capability when dealing with data at different resolutions.

**Figure 11:**
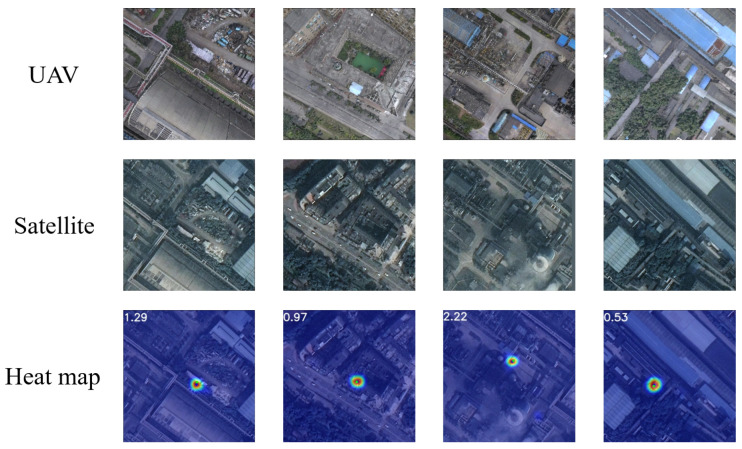
The prediction results are displayed on the heatmaps.

The visualization of the geographic localization results using the VRLM network model is presented in Figure 11. The first row shows the UAV aerial images that require location queries as input to the network, while the second row represents the corresponding satellite images of the query regions. The third row displays the geographic localization results predicted using a heatmap, where the green circles indicate the actual geographic locations, and the blue circles represent the predicted geographic locations based on the output of the localization model. The numerical values in the upper left corner provide a direct measure of the meter-level displacement between the predicted results and the actual labels, vividly illustrating the model’s spatial localization capability.

#### 6.3.2. Comparison of Different Data Sets

**Figure 12:**
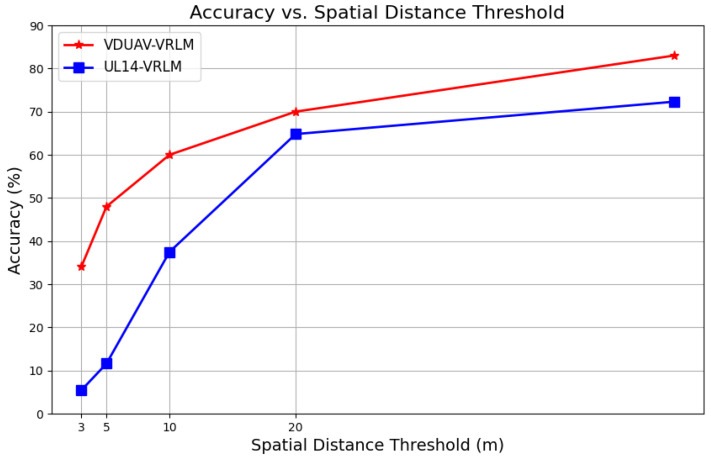
Comparison of localization performance between the UL14 dataset and the VDUAV dataset.

We conducted experiments using the same baseline model and hyperparameter settings on both the UL14 and VDUAV datasets. As shown in Figure 12, the VDUAV dataset significantly outperforms the UL14 dataset at smaller spatial distance thresholds (5 m and 10 m). This discrepancy in accuracy can be attributed to the sampling heights used during data collection. The VDUAV dataset, collected at higher altitudes, captures more spatial features, resulting in richer images and improved localization accuracy. Another potential reason for the performance difference is the method of dataset partitioning. The VDUAV dataset was uniformly sampled across multiple regions and scenarios and then divided into training and test sets at a 3:1 ratio. Due to the dense sampling, some test images might overlap with the training set, enhancing model accuracy. In contrast, the UL14 dataset used 10 universities for the training set and a completely separate set of 4 universities for the test set, ensuring no overlap between the training and test data.

The higher accuracy of the VDUAV dataset at smaller distance thresholds demonstrates its potential for precise localization in scenarios requiring detailed spatial information. These findings highlight the importance of both dataset quality and partitioning strategies in model performance evaluation.

## 7. Controlled Experiment and Analysis

### 7.1. Comparative Experiments on Backbone Networks

**Figure 13:**
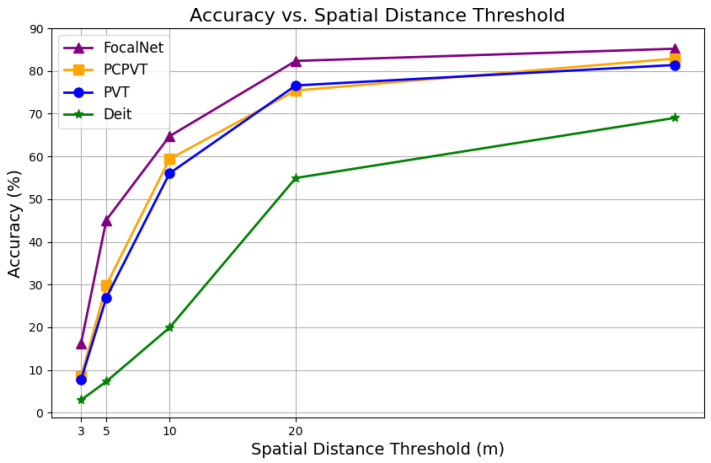
The localization accuracy of the four backbone networks using the MA evaluation metric.

To explore the impact of various backbone networks on the UAV-based geographic localization algorithm, we selected the following mainstream networks: Deit, Pvt, Pcpvt, and FocalNet. These networks were used for feature extraction from both drone and satellite images. As shown in Figure 13 and detailed in Table 5, the experimental results indicate that the Transformer-based Pvt and Pcpvt networks achieve higher localization accuracy compared to the Deit backbone network. The superior performance of Pvt over Deit can be attributed to its pyramid structure, which allows for better multi-scale feature extraction. This capability is particularly beneficial when processing drone and satellite images of different resolutions. On the other hand, Pcpvt combines the local feature extraction ability of convolutional neural networks with the global modeling ability of Transformers, thus showing improved performance in UAV self-localization tasks in complex environments.

The FocalNet network employed in this study introduces a “focal” mechanism, which enhances the model’s ability to focus on specific regions, thereby improving its handling of fine details. This mechanism enables the model to concentrate more on the central areas of UAV images, which leads to improved localization accuracy and robustness. When processing UAV images, FocalNet efficiently captures key feature regions, particularly in geographic localization tasks, outperforming traditional networks that rely solely on global features. This targeted focus on specific feature regions allows the model to maintain stable performance, even in complex terrains and environments, further enhancing its generalization ability and reliability.

### 7.2. Comparative Experiments of Fusion Methods

In fusing the extracted features, this paper considers that the VDUAV dataset contains satellite images of varying scales and complex environmental information, which significantly differs from the drone images input into the network. To address this, we explored using FPN, ASFF, and APFM to fuse the multi-scale features extracted from these images.

**Figure 14:**
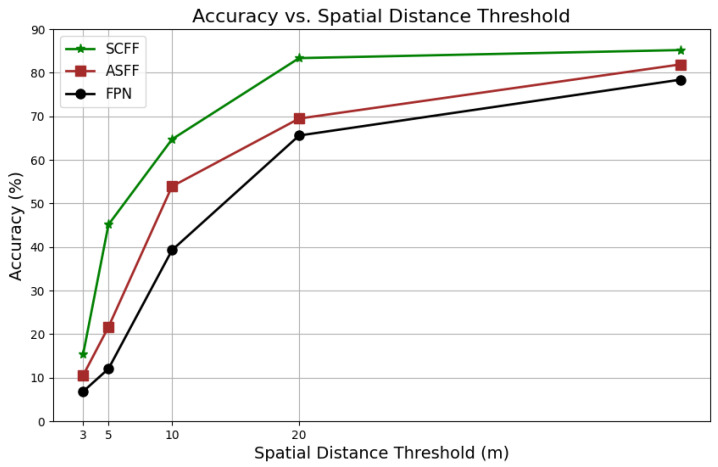
The localization accuracy of the three fusion methods using the MA evaluation metric.

As depicted in Figure 14 and Table 6, the experimental results indicate that the model’s localization performance significantly improved after feature fusion with SCFF, achieving an RDS evaluation metric of 74.13%. In comparison, FPN and ASFF achieved 62.94% and 66.29%, respectively. Specifically, FPN integrates features from different levels through top-down sampling and lateral connections, but its fixed feature fusion approach limits flexibility in handling multi-scale features. Although ASFF enhances flexibility by adaptively selecting and fusing features of different scales through learning weights, it incurs higher computational complexity.

The SCFF (Scale-Constrained Feature Fusion) module combines the strengths of multi-scale fusion from Feature Pyramid Networks (FPN) and adaptive feature fusion from Adaptive Spatial Feature Fusion (ASFF). It processes the three extracted features, U1, U2, U3, by first applying convolution operations to unify them to the same dimension. These are then paired with the corresponding processed features extracted from the satellite images, $S_{1},\;S_{2},\;S_{3}^{*}$, for similarity computation. This process yields adaptive weights α, β, γ for each feature, which are used to perform weighted feature fusion. By doing so, the fused features contain both the shallow spatial information from the low-resolution images and the semantic information from the deeper layers, enhancing the final localization results. This adaptive weighting ensures that the model effectively captures and integrates both detailed local features and global contextual information, resulting in improved accuracy and robustness in UAV localization tasks.

## 8. Conclusions

This study addresses the issue of UAV self-localization in denied environments by focusing on two main aspects: dataset construction and network model design. We introduced the VDUAV dataset and the VRLM cross-view matching model, generating the dataset by automatically capturing images of physical models on a digital twin platform. This approach overcomes the limitations of traditional datasets, which often feature homogenous scenes and are challenging to obtain, making cross-view matching datasets more diverse and representative. On the model front, we leveraged the focal mechanism within the FocalNet network, enabling the model to focus on local features while also integrating global information. This design allows for more effective capture of details and contextual understanding in complex scenes. The multi-scale feature information extracted from the first three stages of the network was processed through the SCFF module, where these features were fused in a multi-scale weighted manner, significantly improving both the model’s operational speed and localization accuracy. Our method achieved remarkable results, with testing on the VDUAV dataset showing an improvement in MA@20 accuracy to 83.35% and an RDS accuracy increase to 74.13%, significantly outperforming previous models.

However, there are still some limitations to our current work. Our model is only capable of determining the latitude and longitude of the UAV’s location and does not yet address altitude localization. Additionally, there is still room for further optimization in both localization accuracy and processing speed. In future research, we aim to address the UAV altitude localization issue and plan to test the approach on real UAV platforms. These efforts will contribute to advancing UAV visual localization technology further.

## Figures and Tables

**Table 1:** Abstract of the existing geographic positioning dataset.

Dataset	Images	Sampling	Target	Source	Platform	Evaluation	Coverage
VDUAV	12.4k	Dense	UAV	Virtual Reality Scene	Virtual Drone-Satellite	RDS&MA	5 provinces
UL14	10k	Dense	UAV	Real Scenes	Drone-Satellite	RDS&MA	Fourteen universities
DenseUAV	20.3k	Dense	UAV	Real Scenes	Drone-Satellite	SDM	Fourteen universities
SUES-200	6.1k	Dense	UAV	Real Scenes	Drone-Satellite	Recall@K & AP	A university
University-1652	50.2k	Discrete	Building	Google Map	Drone-Ground-Satellite	Recall@K & AP	1652 architectures of 72 universities
VIGOR	144k	Discrete	User	Google Map	Ground-Aerial	MA	Four American states
CVUSA	71k	Discrete	User	Google Map	Drone-Satellite	Recall@K	United States

**Table 2:** Dataset collected by UAVs in various regions and multiple scenarios, including geographical environments and sample quantities.

Number	Scenes	Training (Count)	Testing (Count)
1	City	2156	693
2	Plain	1667	574
3	Hill	1196	292
4	Factory	1354	433
5	University	2892	1153
Total	Multiple Scenes	9265	3145

**Table 3:** The ratio of training to testing datasets for UAV images is 3:1, and the ratio of UAV images to satellite images in the training set is 1:12.

Split	UAV (Count)	Satellite (Count)
Train	9265	9265
Test	3145	37,740

**Table 4:** Performance comparison of VRLM method and FPI method on VDUAV dataset.

MODEL	RDS (%)	GFLOPS	Params	MA@5 (%)	MA@10 (%)	MA@20 (%)
FPI	67.07	12.66	42.57	31.81	52.72	71.97
WAMF-FPI	70.48	12.04	34.69	40.27	60.31	78.49
VRLM	74.13	10.28	21.79	45.13	64.72	83.35

**Table 5:** Scoring of RDS based on different backbone networks.

Deit	Pvt	Pcpvt	FocalNet (Our)	RDS (%)
✓				62.94
	✓			66.29
		✓		74.13
			✓	74.13

**Table 6:** Scoring of RDS based on three fusion methods.

FPN	ASFF	SCFF (Our)	RDS (%)
✓			62.94
	✓		66.29
		✓	74.13

## Data Availability

Contact the first/corresponding author please.
